# Olfactory assessment in the Chinese pediatric population

**DOI:** 10.1097/MD.0000000000010464

**Published:** 2018-04-20

**Authors:** Guowei Chen, Hongguang Pan, Lan Li, Jumei Wang, Delun Zhang, Zebin Wu

**Affiliations:** a Department of Otolaryngology, Shenzhen Children's Hospital; b Infirmary of Nanshan Primary School, Shenzhen, Guangdong, China.

**Keywords:** children, odor discrimination, odor identification, olfaction

## Abstract

Young children with olfactory disturbance are sometimes encountered in ENT clinics. We investigated the clinical applicability of olfactory testing to the pediatric population in China.

One hundred and ninety-three healthy children aged 6 to 17 years were enrolled. All participants were asked for demographic information (age, sex, body mass index [BMI], and rating of olfactory function) in a structured questionnaire and underwent olfactory testing including T&T Olfactometer (T&T), odor discrimination (OD), and odor identification (OI) tests of Sniffin’ Sticks.Age had a significant influence on the outcome of olfactory testing, sex, BMI, or self-rating had no influence. Children had better performance on T&T than OI and OD tests of Sniffin’ Sticks.

T&T and Sniffin’ Sticks can be completed by Chinese children. Performance on olfactory tests increased with increasing age. T&T may be more suitable to assess olfactory function in the Chinese pediatric population.

## 1 Introduction

Olfaction is one of 5 basic sensory modalities for human being. It plays an important role in daily human life. It is associated with ingestion, avoiding environmental hazards, and social communication, so its impairment may affect quality of life.^[1,2]^ Olfactory disorders are common and present in 13.5% of the US population.^[3]^ The prevalence of olfactory impairment in children is still unknown. Even olfactory disturbance is less common in children than in adults.^[4]^ However, young children with olfactory dysfunction are sometimes encountered in ENT clinics.^[5]^ More than 200 diseases can lead to olfactory dysfunction.^[6]^ The major causes of olfactory dysfunction for adults in ENT clinics are upper respiratory tract infection, sinonasal diseases, head trauma, and idiopathic disorders.^[7,8]^ The loss of sense of smell for children is commonly caused by adenoid hypertrophy, rhinitis, and rhinosinusitis.^[9–11]^

Several olfactory tests have been developed across the world. In the USA University of Pennsylvania Smell Identification Test is widely used.^[12,13]^ Sniffin’ Sticks olfactory test (Sniffin’ Sticks)^[14,15]^ and T&T Olfactometer (T&T),^[16,17]^ as means of olfactory evaluation, are administered in Europe and Japan separately. In China, both Sniffin’ Sticks and T&T are used to evaluate olfactory function.^[18,19]^ Most studies are focused on olfactory assessment in adults. However, only a few studies on olfactory testing in children have been reported.^[20,21]^ Even no olfactory investigation or relevant studies in the Chinese pediatric population has been reported yet. Therefore, we performed these olfactory tests and investigated the clinical applicability of olfactory testing to the Chinese pediatric population.

## 2 Materials and methods

### 2.1 Participants

One hundred and ninety-three children aged 6 to 17 years were enrolled for assessment of olfactory function. All subjects were in good health and were recruited from Shenzhen Children's Hospital and Nanshan Primary School. Children and their parents/supervisors did not give any history, which could potentially cause olfactory dysfunctions. A structured questionnaire from each child, which included date of birth, sex, height, weight, and how the participant assessed his/her sense of smell (very good/good, average, very bad/bad), was completed. Informed consent from each child and his/her parent or supervisor was obtained. Olfactory function was assessed using T&T (Daiichi Pharmaceutical Co. Ltd., Tokyo, Japan) and Sniffin’ Sticks including odor discrimination (OD) and odor identification (OI) tests (Burghart GmbH, Wedel, Germany). This study compiled with the principles of the Declaration of Helsinki on Biomedical Research Involving Human Subjects and was approved by the Ethical Review Board of Shenzhen Children's Hospital.

### 2.2 T&T

T&T was applied according to the manufacturer's manual. Quantitative analysis was determined by the dilution time of 5 odorants (rose, scorched, rotten, fruit, and stool), each at 8 concentrations (10^−2^–10^5^). Average odor threshold, obtained by dividing the sum of identification threshold for 5 olfactory odorants by 5, was used to assess the degree of olfactory function.^[22]^ The participants’ scores ranged from −2 to 6.

### 2.3 Sniffin’ Sticks

OD and OI tests of Sniffin’ Sticks olfactory test were performed by the standard methods.^[15]^ In OD test, using a 3-alternative forced choice procedure, triplets of pens were presented in a randomized order, with 2 containing the same and 1 a different odorant. Participants had to determine which of 3 pens smelled differently. There were 16 odor identification items in OI test, where each item had 1 correct answer and 3 distractors. Both tests were forced choice paradigms. The participants’ scores on both tests ranged from 0 to 16.

### 2.4 Converted T&T scores for comparison to OD and OI scores

Before comparison to OD and OI scores, T&T scores were transformed into the form of 16 scores. First, 2 was added to T&T score, and the score ranged from 0 to 8. T&T score was different from OD and OI scores, and lower T&T score meant better olfactory function. Second, T&T score was subtracted from 8, and T&T score was changed into the form that higher score meant better olfactory function. Finally, T&T score was multiplied by 2, and the score ranged from 0 to 16. New T&T scores were used to compare with OD and OI scores.

### 2.5 Statistical analysis

Results were performed using SPSS 17.0 for Windows (SPSS Inc, Chicago, IL). Data were presented as mean and standard deviation for continuous measures. Group comparisons were conducted using *t* test and analysis of variance. The level of statistical significance was set at 0.05, and all reported *P* values are 2-tailed.

## 3 Results

In total, 193 children (98 males, 95 females) with a mean age of 11.56 ± 3.04 years (ranged 6–17 years) were enrolled in the study. All participants completed T&T, OD, and OI tests. The participants were separated into 3 age groups (A–C): group A, 6 to 9 years; group B, 10 to 14 years; and group C, 15 to 17 years.

**Figure 1:**
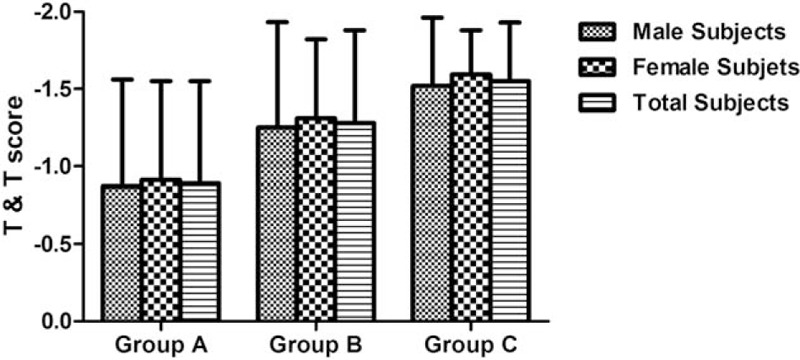
T&T Olfactometer (T&T) scores for participants in different age and sex groups.

**Figure 2:**
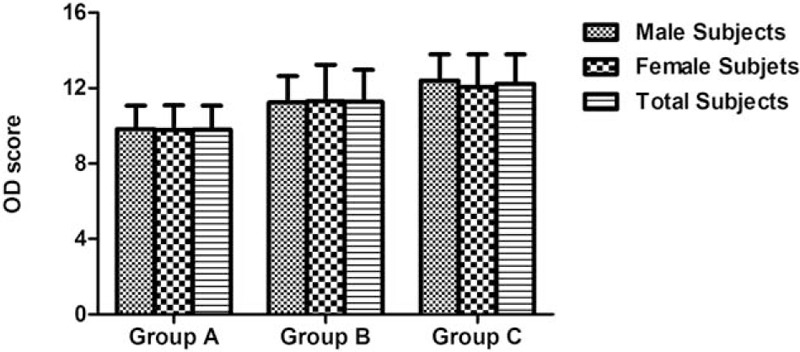
Odor discrimination (OD) scores for participants in different age and sex groups.

**Figure 3:**
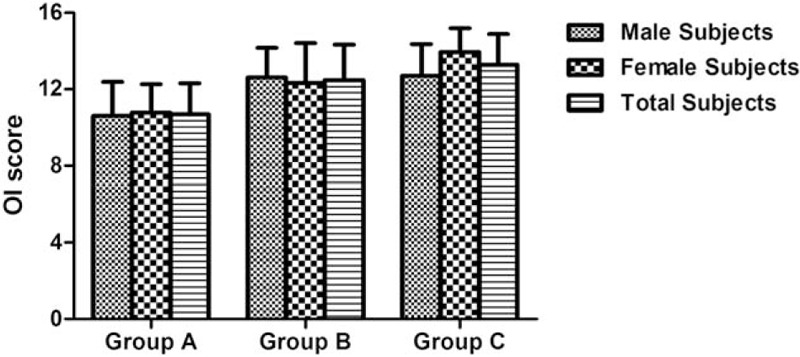
Odor identification (OI) scores for participants in different age and sex groups.

No significant main effect of the factor “sex” was found for the scores on T&T (*F* = 0.379, *P* = .539), OD (*F* = 0.195, *P* = .660), or OI (*F* = 1.916, *P* = .168). There was no significant interaction between factors “sex” and “age group” for the scores on T&T (*F* = 0.007, *P* = .993), OD (*F* = 0.227, *P* = .797), or OI (*F* = 2.714, *P* = .069), as shown in Table 1 and Figures 1 to 3.

**Table 1:**
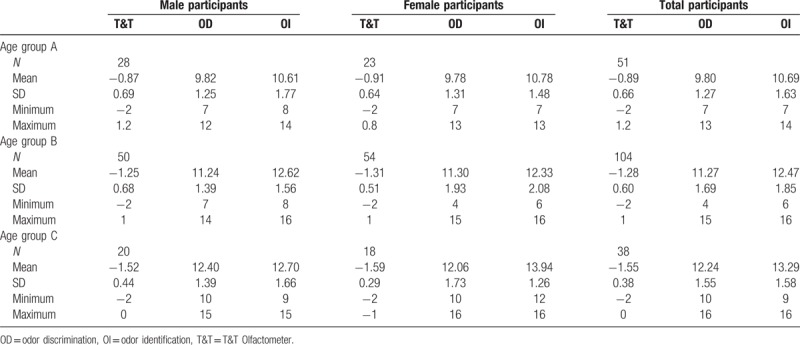
Descriptive statistics of olfactory performance for participants separated into sex groups.

In total participants, comparison of T&T, OD, and OI scores obtained from age groups A to C revealed significant differences for the scores on T & T (*F* = 15.057, *P* < .001), OD (*F* = 28.336, *P* < .001), and OI (*F* = 27.794, *P* < .001). Bonferroni post-hoc tests showed significant differences between age groups for the scores on T&T, OD, and OI. Significant main effect of the factor “age group” was found for the scores on T&T (*F* = 14.701, *P* < .001), OD (*F* = 27.651, *P* < .001), and OI (*F* = 28.304, *P* < .001), as shown in Table 1 and Figures 1 to 3.

No significant difference was revealed for the scores on T&T (*F* = 0.866, *P* = .422), OD (*F* = 0.236, *P* = .790), or OI (*F* = 1.548, *P* = .215) in body mass index (BMI) groups, as shown in Table 2.

**Table 2:**
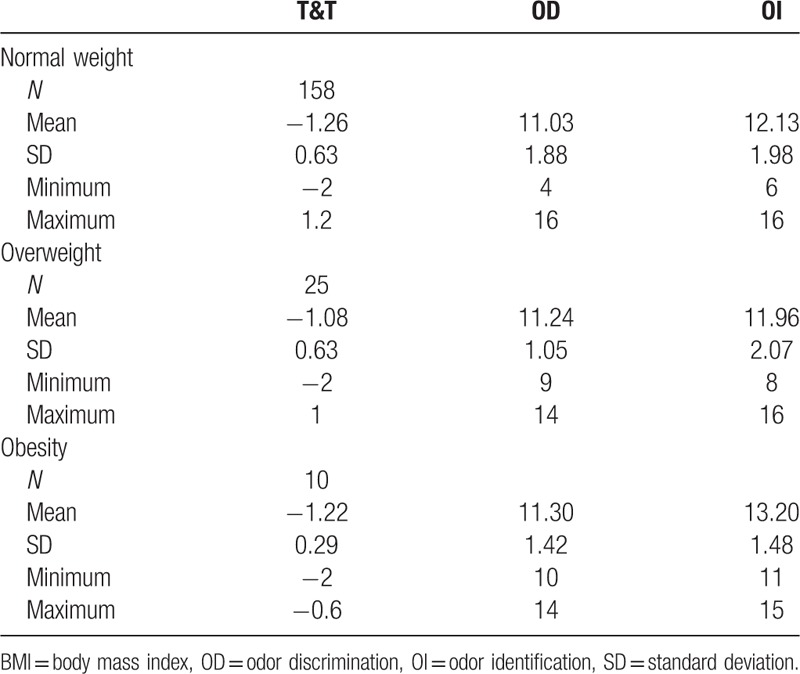
Descriptive statistics of olfactory performance for participants separated into BMI groups.

No significant difference was revealed for the scores on T&T (*F* = 0.825, *P* = .440), OD (*F* = 0.142, *P* = .867), or OI (*F* = 2.323, *P* = .101) in self-rating groups, as shown in Table 3.

**Table 3:**
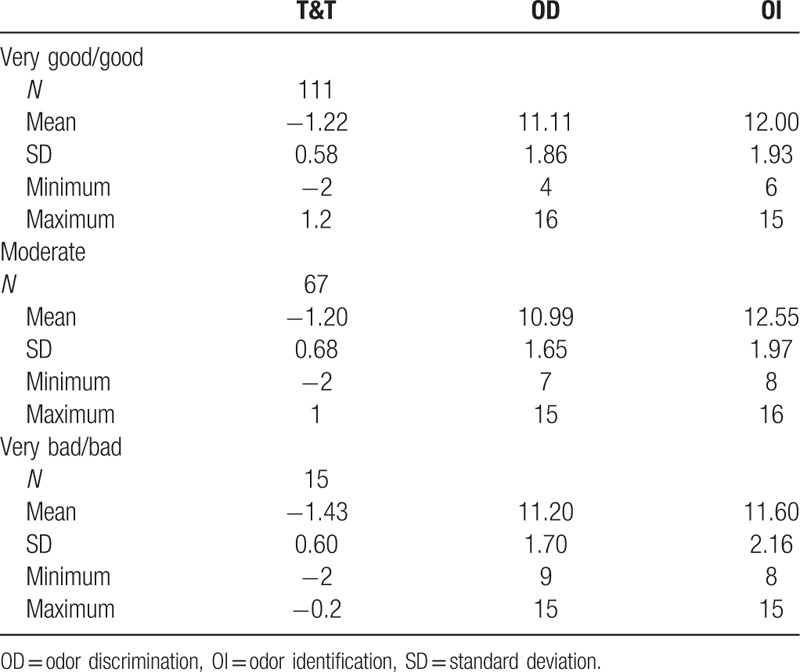
Descriptive statistics of olfactory performance for participants separated into self-rating groups.

**Figure 4:**
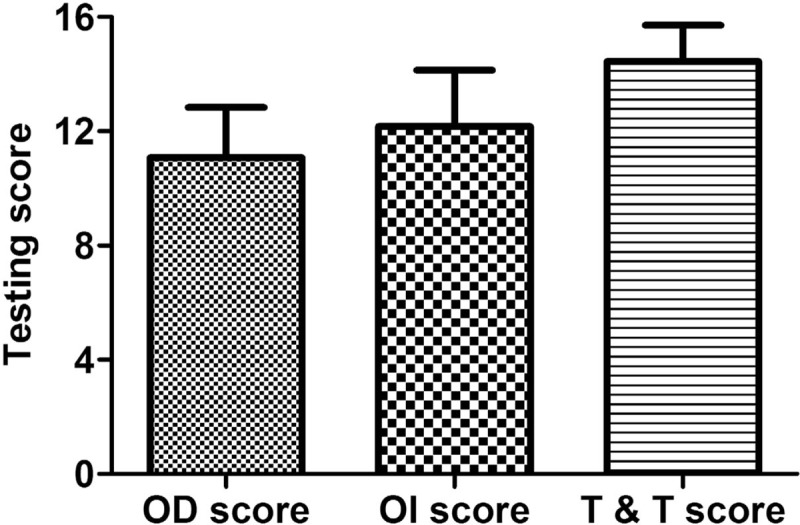
Testing scores comparing olfactory tests.

Before comparison to OD and OI scores, T&T scores were transformed into the form of 16 scores. The overall mean score for OD was 11.07, for OI was 12.16, and for T&T was 14.45. Comparison to OI, OD, and T&T scores indicated a significant difference (*F* = 199.816, *P* < .001), and Bonferroni post-hoc tests showed a significant difference between 3 groups, as shown in Figure 4.

## 4 Discussion

T&T and Sniffin’ Sticks are modern olfactory tests that have been thoroughly validated for adults. In some studies Sniffin’ Sticks has been applied to investigate olfactory function in children.^[20,21]^ Compared with visual and hearing loss, olfactory impairment is generally ignored for lack of awareness. It has been reported that poor olfactory performance is shown in children younger than 6 years.^[23–25]^ Low attention span, linguistic development, and lack of odor experience may contribute to the result that the clinical diagnosis of olfactory disorders in children is difficult.^[24,26,27]^ The factors listed above can lead to the fact that no relevant studies assessing olfactory function in Chinese children had been reported. We used T&T, OD, and OI tests to assess olfactory function of children in the present study. These tests were successfully administered to the enrolled children between 6 to 17 years of age. We did not include children younger than 6 years because previous studies have shown that children younger than 6 had poor olfactory performance.^[23–25]^ Our study aims at investigating the clinical application of olfactory testing to the Chinese pediatric population, not at providing the normative data as a clinical guide.

It is well known that sex-related differences in olfactory tests exist in adults. However, most studied have reported that there is no significant effect of sex on the performance of olfactory tests in children.^[15,23,28]^ In line with these reports, olfactory performance between boys and girls is not significantly different in the present study. It was also reported that olfactory performance of girls was better than boys.^[20]^ Hormonal effects, verbal skills, and/or congenital factors may contribute to the sex-related differences in adults.^[15]^ It is still a matter of debate whether these factors play a role in children. We cannot determine an effect of sex on olfactory function. Maybe this sex difference is so subtle that olfactory testing cannot detect such a difference. Also, olfactory testing is at the similar level of difficulty for boys and girls. Even boys and girls having the similar cognitive abilities may contribute to the negative result.

Our study shows that olfactory performance in children increased with age, which is in line with previous studies.^[20,21,23,24,29]^ We created 3 age groups, according to children's olfactory performance. Each group differed significantly in testing scores from the other 2. Within each group, testing scores were not affected by the factor “age.” Several factors may contribute to the positive result, which includes verbal abilities, familiarity with odors, and cognitive capacity to identify certain odors.^[28,30]^ These factors are associated with cognitive level and seem to develop with age. Cognition is known as a significant determinant of olfactory performance other than age.^[31]^ Thus, the effect of age on olfactory performance is thought to be the effect of age on the cognitive abilities involved in olfactory testing.

Obese children have been reported to have worse olfactory performance, which is linked to metabolic disturbances.^[32]^ In the present study, we cannot determine an effect of BMI on olfactory function, which is in accordance with the previous work.^[20,29]^ One of the possible reasons explaining the negative result is the smaller sample size of the obese children. Only 7 of 193 children were considered to be obese, according to BMI reference norm for Chinese children and adolescents.^[33]^

Self-ratings of olfactory function are not always consistent with the results of smell testing. Some studies reported that self-evaluated olfactory performance was not related to olfactory testing in normosmic adults.^[34,35]^ Opposite opinion was also reported.^[28]^ In the present study, we show that self-evaluated olfactory function is not related to olfactory testing in normosmic children, which is in line with the previous study.^[28]^ Cognitive abilities are less developed in children. To some extent, it was difficult for them to rate their olfactory function on their own. It may contribute to the result that children were not able to accurately evaluate their own olfactory sensitivity. Self-assessments of olfactory function are rather unreliable in children.

Overall, our results show that these tests can be applied to the pediatric population in China. In the present study, children have better performance on T&T than OD and OI tests of Sniffin’ Sticks. Familiarity with odors varies from different cultures.^[36,37]^ Chinese and Japanese share similar culture; thus, odorants on T&T may be more easily recognized by the participants. This may be the reason that children have better olfactory performance on T&T. Limitations of the present study are that cognitive tests were not conducted and children with olfactory disturbances were not included. Therefore, the influence of cognition on olfactory ability and the olfactory performance of children with olfactory dysfunction could not be observed. Further research in these areas is warranted.

## 5 Conclusions

T&T and Sniffin’ Sticks can be completed by children aged between 6 and 17 years in China. Performance on olfactory tests increased with increasing age. T&T may be more suitable to assess olfactory function in Chinese pediatric population.

## Author contributions

**Conceptualization:** Guowei Chen, Hongguang Pan.

**Data curation:** Guowei Chen, Hongguang Pan.

**Formal analysis:** Guowei Chen, Hongguang Pan.

**Funding acquisition:** Hongguang Pan.

**Investigation:** Guowei Chen, Hongguang Pan, Lan Li, Jumei Wang, Delun Zhang, Zebin Wu.

**Methodology:** Guowei Chen, Hongguang Pan, Lan Li, Delun Zhang, Zebin Wu.

**Project administration:** Guowei Chen, Hongguang Pan.

**Resources:** Guowei Chen, Hongguang Pan, Jumei Wang, Zebin Wu.

**Software:** Guowei Chen, Hongguang Pan, Zebin Wu.

**Supervision:** Guowei Chen, Hongguang Pan, Lan Li.

**Validation:** Guowei Chen, Hongguang Pan, Lan Li.

**Visualization:** Guowei Chen, Hongguang Pan, Lan Li.

**Writing – original draft:** Guowei Chen, Hongguang Pan.

**Writing – review & editing:** Guowei Chen, Hongguang Pan.
